# Fatal hemopericardial tamponade due to primary pericardial mesothelioma: a case report

**DOI:** 10.1186/1746-1596-4-44

**Published:** 2009-12-09

**Authors:** Daniel C Lingamfelter, Dominick Cavuoti, Amy C Gruszecki

**Affiliations:** 1 Jackson County Medical Examiners Office, Kansas City, Missouri, USA; 2 University of Texas Southwestern Medical Center, Dallas, Texas, USA; 3 University of Texas Southwestern Medical Center, and Medical Examiner, Southwestern Institute of Forensic Sciences, Dallas, Texas, USA

## Abstract

**Background:**

Primary mesothelioma of the pericardium comprises less than 1% of all mesothelioma cases. Its typical presentation is insidious, with nonspecific signs and symptoms, and usually results in heart failure due to cardiac tamponade, either by a serous effusion or by direct tumorous constriction of the heart. With the exception of several case reports, the outcome is uniformly fatal, and patients typically die within six months of diagnosis.

**Case presentation:**

A 45-year-old African American female presented to the emergency department with several days of dizziness, difficulty walking, and low blood pressure. The patient suddenly suffered cardiac arrest, and her death was pronounced. The medical examiner assumed jurisdiction of the case due to the sudden death nature of the case without known medical history. At autopsy, a one-liter hemopericardium was present, and the pericardial sac was thick, granular and adhesed to the heart, suspicious for pericarditis. Microscopic examination of the pericardial tissue instead led to a diagnosis of primary pericardial mesothelioma.

**Conclusion:**

Our case demonstrates a pericardial mesothelioma initially masquerading grossly as pericarditis. Microscopic examination of any grossly abnormal pericardial tissue therefore may be warranted so that a neoplastic disease process does not go undetected. Additionally, of the approximately 200 such tumors reported in the medical literature, a case demonstrating marked hemopericardium and resulting in sudden death has not been described until now.

## Background

Fortunately, mesotheliomas are very uncommon tumors, with an incidence of approximately one per million. Such lesions arising from the pericardium constitute only 0.7% of all cases and therefore represent an extremely rare entity [1]. This neoplasm typically presents insidiously with rather nonspecific signs and symptoms including dyspnea, fever, chest pain, and weight loss. Common clinical manifestations include constrictive pericarditis, pericardial effusion, cardiac tamponade, and eventual heart failure usually stemming from either physical compression or myocardial tumorous infiltration. To date, there are no reported cases in the medical literature describing a pericardial mesothelioma causing marked hemopericardium, and resulting in sudden death. Herein, we report such a case.

## Case Presentation

**Figure 1:**
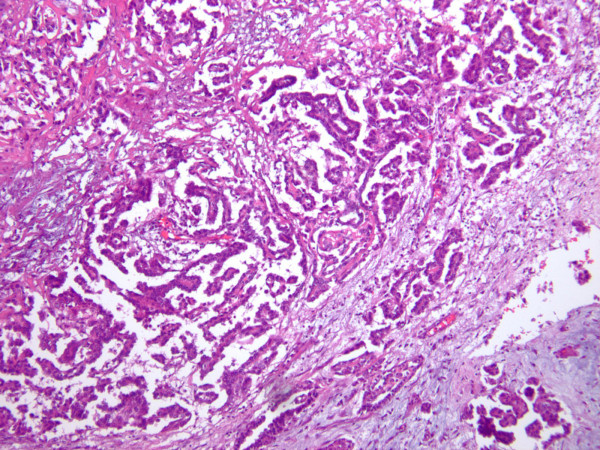
**The epicardial surface shows numerous neoplastic epithelioid cells**. In some areas, the tumor cells form gland-like structures and papillary structures (H&E, ×40).

**Figure 2:**
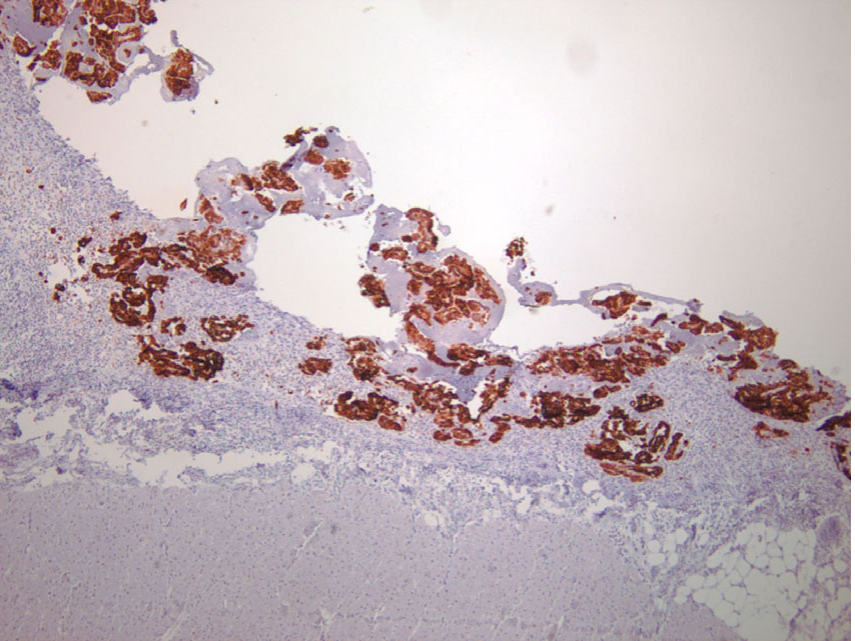
**The cell membranes of the neoplastic cells react strongly and diffusely with antibodies to CK 5/6 (×100)**. The myocardium can be observed lying just underneath the band of neoplastic tissue.

**Figure 3:**
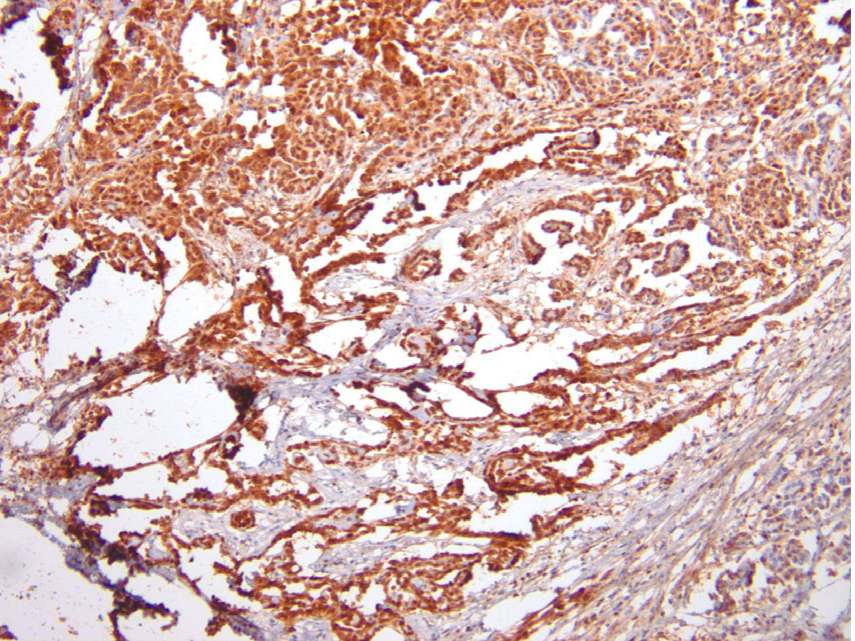
**Immunohistochemistry for calretinin displays marked nuclear and cytoplasmic positivity for the tumor cells (×100)**.

A 45-year-old African American female presented to the emergency department (ED) with a new onset of dizziness, difficulty walking, and low blood pressure. Her past medical history was significant for hypercholesterolemia, hypertension, and a recent urinary tract infection. She smoked tobacco but used neither alcohol nor illegal drugs. There was no history of asbestos exposure or recent viral illness. Physical examination was unremarkable. Lab work was performed and showed normal results, so the patient was released. The following day, she returned to the ED once again, and reported that her symptoms had escalated significantly. Soon thereafter the patient developed multiple organ failure with subsequent cardiac arrest, passing into pulseless electrical activity, and her death was pronounced. The initial significant autopsy finding was the discovery of approximately one liter of blood within the pericardial cavity. The pericardial sac was focally adhesed to the surface of the heart. The pericardial surface of the heart was markedly granular and thickened. An area on the pericardium of the anterior left ventricle was focally disrupted without frank tearing or laceration and blood was observed to ooze through this defect. Gross photographs were not taken due to the initial impression of classic pericarditis. The remainder of the postmortem examination was negative for grossly suspicious malignancy, including pleural, pulmonary and lymphoreticular involvement. No abdominal or pelvic pathology was identified. In particular, there was no omental or peritoneal caking, and no ovarian, renal, or hepatic masses were identified. Microscopic examination of tissue sections taken from the epicardium revealed dense lymphocytic infiltrates admixed with numerous neoplastic, polygonal cells with abundant cytoplasm and enlarged, irregular nuclei containing conspicuous nucleoli. Some of these tumor cell clusters formed gland-like formations and papillary structures (Figure 1). Abundant necrosis and marked desmoplasia were present. Scattered karyorrhectic cells were present while mitotic figures were not identified. There was only mininal tumoral infiltration into the underlying myocardium. Pericardial calcifications were not appreciated. Immunohistochemical stains were performed on the paraffin-embedded epicardial tissue, resulting in marked positivity for pancytokeratin, CK5/6 (Figure 2) and calretinin (Figure 3). A diagnosis of primary pericardial mesothelioma was thereby confirmed. The cause of death was reported as cardiac tamponade due to pericarditis due to mesothelioma of the pericardial sac.

## Discussion

Several studies demonstrate the infrequent nature of primary pericardial mesotheliomas. A ten-year focused autopsy review of malignant tumors by Mukai, et al., recovered a single case out of a total of 2,649 cases while a larger 500,000 case study provided an incidence of less than 0.0022% [2,3]. Of the approximately 200 reported cases in the medical literature, only one of four was diagnosed while the patient was still alive [4]. And, in contrast to pleural mesotheliomas, there is no strong correlation between pericardial mesothelioma and asbestos exposure.

Perhaps the reason for the lesion's low detection rate derives from its typical clinical presentation, a group of nonspecific signs and symptoms of insidious onset related to declining cardiac function - dyspnea, chest pain, fever, and weight loss - and manifesting as restrictive pericarditis, pericardial effusion, and cardiac tamponade [5]. Local vascular invasion may result in cerebral ischemia, myocardial infarction, and superior vena cava syndrome [1].

The usually progressive disease process appears to be the only positive characteristic regarding this tumor, in order that the patient's condition be diagnosed and afforded at least some measure of treatment. Unfortunately, the overall survival rate is less than six months, and treatment is focused on palliation, including such procedures as partial pericardectomy for relief of cardiac tamponade [6]. Various chemotherapeutic agents have shown little efficacy; high-dose methotrexate has shown the only response rate over 20% [7]. Pharmocologic therapies including lovastatin and a Simian virus 40-based vaccine (SV40) are still unproven [7]. The authors are unaware of any data regarding the use of the SV40 vaccine against pericardial mesotheliomas.

Over the last decade, the SV40 virus has stepped into the forefront of suspected agents in the pathogenesis of mesothelioma [8]. The virus has been shown to cause mesothelial tumorigenesis in a number of animal models, and specific SV40 DNA sequences have been discovered in malignant pleural mesotheliomas [7]. Carbone, et al., report that at least 60% of mesotheliomas in the United States contain and express SV40, which results in suppression of tumor suppressors p53 and Rb, thereby leading to tumor development [9].

The typical gross pathologic features of pericardial mesothelioma are that of a tan, variably nodular to solid thickening of the pericardium, sometimes extending into the aorta and pulmonary trunk. Epicardial invasion has been reported, but deep invasion of the myocardium is not common [1]. Histologically, three different subtypes - epithelial, sarcomatoid, and mixed (75% of cases) - have been described.

The usual immunohistochemical positivity of pleural mesotheliomas for cytokeratin (CK 5/6), calretinin, and HBME-1 with negative staining for carcinoembryonic antigen (CEA), B72.3, and Leu-M1, holds true for the pericardial variety. Kayser, et al., discovered that a combination of probes with antibodies against carrier-immobilized ganglioside GM1, hyaluronic acid, calretinin, and HBME-1 was highly sensitive and specific for distinguishing mesothelioma from metastatic carcinomas, as the markers for ganglioside GM1 and hyaluronic acid showed the highest sensitivity and specificity for mesothelioma. In contrast, CEA and N-acetyl-D-glucosamine-bearing neoglycoprotein displayed high sensitivity and specificity for metastatic carcinomas [10].

In this case, the original diagnosis based upon the gross examination was that of a diffuse, marked pericarditis with associated profound hemopericardium. However, upon initial microscopic examination, while lymphocytic pericarditis was in fact present, the more significant neoplastic entity was additionally identified. Pericarditis, especially the fibrinous variety, can grossly mimic pericardial mesothelioma, as it may exhibit thickening and a fine granularity of the pericardium. A host of tumors may also enter the differential diagnosis. While primary pericardial neoplasms including angiosarcoma, teratoma, and fibroma can occur, they are rare also. More commonly encountered are metastatic tumors originating from lung, breast, skin (melanoma), as well as lymphomas and leukemias [11].

When confronted with the finding of hemopericardium during an autopsy, the forensic pathologist may have a number of different etiologies to entertain. Altun, et al., encountered 40 deaths related to hemopericardium and concluded that the overwhelming majority of these deaths were trauma-related [12]. Stabbings accounted for half of the cases while gunshots and shotgun wounds collectively comprised approximately 40% of the deaths. Only two cases were natural demises, although the specific causes of death were not provided.

Several relatively common causes of hemopericardium encountered during autopsy include type A aortic dissections and ruptured myocardial infarctions. A number of other different etiologies, albeit rare, have been reported including vascular lesions (angiosarcoma, hemangioma), anticoagulation therapy, acute bacterial pericarditis, Takayasu aortitis, and rheumatoid arthritis [13-16].

## Conclusion

This challenging autopsy case initially presented as a diffuse, fibrinous pericarditis causing considerable hemopericardium and resulting in sudden death. Histologic and immunohistochemical analyses were essential in elucidating, and confirming, the presence of a primary pericardial mesothelioma. This case, among others encountered in our review of the literature, questions the accuracy of a diagnosis of pericarditis based solely on gross inspection. Without a microscopic examination of grossly abnormal pericardial tissue, a more significant disease process could go undetected. This includes a rare, but possible, neoplastic process.

## Consent

Consent was obtained for publication of this case report and accompanying images. A copy of the consent is available for review by the Editor-in-Chief of this journal.

## Competing interests

The authors declare that they have no competing interests.

## Authors' contributions

DL constructed the majority of the manuscript. DC provided the histological interpretations and contributed to manuscript proofreading. AG provided the case history and autopsy findings and contributed to manuscript proofreading.
